# The availability of health information system for decision-making with evidence-based medicine approach-a case study: Kermanshah, Iran

**DOI:** 10.1016/j.dib.2018.05.122

**Published:** 2018-05-24

**Authors:** Ameneh Safari, Yahya Safari

**Affiliations:** Department of Radiology, Paramedical School, Kermanshah University of Medical Sciences, Kermanshah, Iran

**Keywords:** Evidence-Based medicine, Hospital information system, Clinical decision

## Abstract

Evidence-based medicine (EBM) is defining proper and wise use of the best evidence in clinical decision for patient׳s care. This study have done with the aim of evaluating health information system for decision-making with EBM approach in educational hospital of Kermanshah city. The statistical population include all the specialist and specialty, and also head nurses of educational hospitals in Kermanshah city. The data collected by researcher made questionnaire. The content validities of the questionnaire were confirmed by experts to complete the questions of the questionnaire. Then, the reliability of the questionnaire was evaluated using the Cronbach׳s alpha coefficient. The results have showed that the accessibility rate to the internet sources is in desirable level. The results have showed that there was a significant difference at least in one group between the availability of hospital information system EBM establishment in terms of accessing to the internet based data, according to the academic major (*P = 0.021*). The sufficiency of hospital information system in evidence-based medicine establishment in terms of necessary knowledge for implementing it according to the educational major have showed a significant statistical difference at least in one group (*P = 0.001*). Kermanshah׳s hospital have a desirable condition in terms of accessibility to the internet sources, knowledge of EBM and its implementation which this have showed the availability of desirable platform for decision-making with the EBM approach. However, it is better to implement regulate educational periods for educating the doctors and nurses in order to reach practical implementation of the EBM approach.

**Specifications Table**

**Table:** 

Subject area	Medicine
More specific subject area	Health management
Type of data	Tables
How data was acquired	In this descriptive cross-sectional study, the The availability of health information system for decision-making with evidence-based medicine approach investigated. The statistical population of this study consisted of all the specialist and specialty, and also head nurses of educational hospitals in Kermanshah city. The data collection was done by the researcher made questionnaire and the collated raw data analyzed by SPSS software(Ver.21).
Data format	Raw, analyzed
Experimental factors	The content validities of the questionnaire were confirmed by experts to complete the questions of the questionnaire. Then, the reliability of the questionnaire was evaluated using the Cronbach׳s alpha coefficient [1], [2], [3], [4], [5], [6].
Experimental features	For comparison of demographic properties of responders based on the study׳s aims, for data normal distribution, the ANOVA parametric test and for non-normal, the non-parametric Kruskal-Wallis test (at significance level of P <0.05) used.
Data source location	Kermanshah, Iran
Data accessibility	Data are included in this article

**Value of the data**

• Evidence-based medicine (EBM) is defining proper and wise use of the best evidence in clinical decision for patient׳s care [1], [2], [3]. In fact, evidence-based medicine is a combination of the best evidence, which obtained from the researches of clinical experiences and patient׳s desire [4], [5] and use of the best evidences is its highest benefits [6].
• One of the important cases in clinical decision were evaluation and critical judgment of observation and improper use of evidence could have led to patient׳s death. Therefore, it is necessary that the doctors could search and retrieve their proper evidence and combine it with their experiences and applied it for their patients.
• Various studies have showed that the doctors have low knowledge of EBM and do not have the necessary skills for applying this method. While the knowledge and skills, which have related to EBM, were the important factors of using this process [1], [7], [8], [9]. Also it have presented that the Iran hospital information system do not have enough pieces for accessing to the health information based on the internet and reference database.
• According to the above points and the necessity of knowledge about EBM, also applying it in treatment and care process, this study have done with the aim of evaluating the health information system for decision-making with EBM approach in educational hospitals of Kermanshah city.
• This is the first study in Kermanshah, and its data can be useful for decision making. In addition, the present study data can be useful for future similar studies in other locations of Iran.
• Based on the obtained data of this study, the Kermanshah hospitals have proper status in terms of internet resources accessibility, having proper awareness about EBM and its implementation.

## 1 Data

As it have mentioned in Table 1, 46 person (57.5%) head nurses, 31 person (38.7%) specialist and 3 person (3.8%) specialty have participated in this study.

**Table 1:** Demographic properties of studied doctors and head nurses.

**Variable**		**Frequency (%)**
Gender	Woman	36 (55)
	Man	44(45)
Head nurses		46(57.5)
Specialist physician		31(38.8)
Specialty physician		3(3.8)

Based on the results of Table 2, among the related questions of accessibility rate to internet resources, the highest average score (4.11) was for accessing to public internet site and the least average score (3.17) was for accessing the internet to the patients list.

**Table 2:** The accessibility rate of doctors and head nurses of Kermanshah medical science hospital to the internet sources.

**The accessibility rate**								
**Frequency: Number (Percent)**								**S.D**
**Variables**	**Always**	**Often**	**Sometime**	**Seldom**	**Never**	**Mean**		
Internet sources	30 (37.5)	31 (38.8)	16 (20)	2 (5.5)	1 (1.2)	4.08		0.88
Internet public site	34 (37.5)	28 (38.8)	13 (16.2)	3 (3.8)	2 (2.5)	4.11		0.98
Internet site with health information	28 (35)	32 (40)	16 (20)	2 (2.5)	2 (2.5)	4.02		0.94
Internet site with treatment information	26 (32.5)	34 (42.5)	15 (18.8)	4 (5)	1 (1.2)	4		0.91
Possibility of search with searching motor	16 (32.5)	29 (36.2)	20 (25)	4 (5)	1 (1.2)	3.93		0.94
Search with special searching method	19 (23.8)	21 (26.2)	23 (28.8)	11 (13.8)	6 (7.5)	3.87		1.03
Encyclopedias and medical newsletters	17 (21.2)	23 (28.8)	30 (37.5)	6 (7.5)	4 (5)	3.53		1.06
Bank disease cases	21 (26.2)	28 (25)	21 (26.2)	7 (8.8)	3 (3.8)	3.74		1.03
Patient list	10 (12.5)	23 (28.8)	23 (28.8)	20 (25)	4 (5)	3.17		1.1
Last updated of medical Books	12 (15)	28 (35)	15 (18.8)	19 (23.8)	6 (7.5)	3.25		1.2
Journals	12 (15)	23 (28.8)	24 (30)	16 (20)	5 (6.2)	3.36		1.15
Drug information bank	12 (15)	19 (23.8)	24 (30)	16 (20)	5 (6.2)	3.25		1.13
Drug bank	14 (17.5)	21 (26.2)	30 (37.5)	12 (15)	2 (2.5)	3.41		1.03

As it have shown in Table 3, among the questions which have related to the knowledge rate of doctors and head nurses of affiliated hospitals of Kermanshah Medical Science University, the most average score (3.12) was for the familiarity level with digital library and EBM and the least average score (3.73) was for passing EBM period.

**Table 3:** The knowledge rate of doctors and head nurses of KUMS’s hospital about EBM.

**Knowledge level**							
**Frequency: Number (Percent)**							
Familiarity level with digital library	4 (5)	21 (26.2)	40 (50)	11 (13.8)	4 (5)	3.12	0.89
Familiarity level with evidence-based medicine	6 (7.5)	18 (22.5)	40 (50)	12 (15)	4 (5)	3.12	0.93
Passing evidence based medicine period	6 (7.5)	18 (22.5)	40 (50)	12 (15)	4 (5)	2.72	1.21
Educating the use of hospital information system	4 (5)	15 (18.8)	31 (38.8)	17 (21.2)	13 (16.2)	2.75	1.09

Based on the mentioned results of Table 4, among the related questions to the level of EBM implementation in doctors of affiliated hospitals of Kermanshah Medical Science University the highest average score (3.76) was for EBM judgment and the least average score (3.42) was for EBM clinical act.

**Table 4:** The level of EBM implementation in Kermanshah Medical Science University’s hospitals.

**The level of evidence-based medicine implementation**							
**Frequency: Number (Percent)**						**Mean**	**SD**
**Variables**	**Always**	**Often**	**Sometime**	**Seldom**	**Never**		
Highlighting the main question with appropriate key-words	18 (22.5)	27 (33.8)	25 (31.2)	6 (7.5)	4 (5)	3.61	1.07
Searching resources for reaching the evidences	14 (17.5)	25 (31.2)	31 (38.8)	7 (8.8)	3 (3.8)	3.5	1.00
Measurement to identify an interpreting evidence	15 (18.8)	24 (30)	35 (43.8)	3 (3.8)	3 (3.8)	3.56	0.96
Evidence-based clinical action	9 (11.2)	31 (38.8)	29 (36.2)	7 (8.8)	4 (5)	3.42	0.97
Evidence-based decision	16 (20)	27 (33.8)	33 (41.2)	2 (2.5)	2 (2.5)	3.66	0.912
Evidence-based judgment	15 (18.8)	41 (51.2)	16 (20)	6 (7.5)	2 (2.5)	3.76	0.93

For evaluating the sufficiency of hospital information system in establishment of EBM in terms of accessing the internet-based health information, according to the academic major, for normality of data distribution, the Lon test results have showed that the data have normal distribution (*P > 0.05*). Based on this fact the ANOVA test result have showed that at least in one group there was a significant difference between the sufficiency of hospital information system in establishment of EBM in terms of accessing the internet based health information due to the academic major (*P = 0.02*) (Table 5). For evaluating the fact that which majors are different, the Bonferroni post-hoc test have used and the results of this test have showed that there was a significant difference between the specialist of cardio vascular disease with head nurse, and infectious specialist with cardiovascular specialist.

**Table 5:** The ANOVA results of EBM establishment in terms of accessing to the internet based health information due to the academic major of responders.

**Changes resources**	**Sum of squares**	**Mean of squares**	**F**	***P***
Among a group	2712.77	339.09	3.40	0.02
Inter group	6970.56	99.58		
Total	9683.34	–		

For evaluating the sufficiency of hospital information system and establishment of EBM in terms of necessary knowledge for its implementation, according to the academic major, the Lon test results have showed that variances were homogenous (*P > 0.05*). based on the ANOVA of the academic major and the system sufficiency in terms of knowledge and EBM implementation there was a statistical significant difference at least in one group (*P < 0.001*) (Table 6).

**Table 6:** The ANOVA results of EBM establishment in terms of accessing to the internet based health information and knowledge necessary for the implementation of EBM.

**The evaluated variable**	**Average difference**	**t**	***P***
Accessing internet resources	0.18	0.071	.943
Having the knowledge of evidence-based medicine and its implementing	1.69	1.52	0.131

Based on the Bonferroni post-hoc test there was a significant statistical difference between the internal and cardiovascular specialist, internal and gynecologist specialist, cardiovascular and head nurses, internal specialist and other academic majors.

For evaluating the sufficiency of the hospital information system in establishment of EBM in terms of accessing the internet health information and having necessary knowledge for implementing EBM, according to the gender, first the variances equality have investigated and they were equal. Then the t-test results have showed that there was not any significant statistical difference between the men and women participants of the research (*P > 0.05*).

## 2 Study design, materials and methods

This study have done with descriptive cross-sectional method in 2017. All the specialist, specialty and head nurses of various parts in affiliated hospital to the Kermanshah University of Medical Science (KUMS) have included the statistical population. Sampling of this research have done with random sharing method. At the beginning of the study the number of specialist and nurses of various parts of educational hospitals in Kermanshah city have estimated and after that with the proportion of the statistical population and statistical samples each hospital take a share of them randomly. By using the krejcie-morgan table, the samples volume have calculated. By using the questionnaire, the data have collected. The content validities of the questionnaire were confirmed by experts to complete the questions of the questionnaire. Then, the reliability of the questionnaire was evaluated using the Cronbach׳s alpha coefficient [10], [12], [13], [14], [15], [16]. The formal and content validity of this questionnaire have validated by the experts of the medical and care information management major, which have some expertise in the field of EBM and some related experts with implementing EBM in Imam Reza educational hospital of Kermanshah. The reliability of the questionnaire have estimated by the internal consistency coefficient also the cronbach׳s alpha have calculated 0.81 The first part of the questionnaire have related to demographic properties of the participants. The second part includes 24 questions, which classified in four parts. 20 questions have presented in 5 Likert range (always=5 to very low=1) and 4 other question in 5 Likert range (very high=5 to very low=1). The designed questionnaire have distributed among all the study׳s sample and all the affiliated hospitals to KUMS and they have asked to answer the questions.

At the end the collected data of the questionnaire have enter the SPSS.ver21 software and by using the descriptive statistic (the frequency, mean and standard deviation) the descriptive tables have presented. Also after evaluating the normality by Kolmogorov Smirnov Test, the t-test used for comparison of the means with the hypothesis means. Also for comparison of demographic properties of responders based on the study׳s aims, for data normal distribution, the ANOVA parametric test and for non-normal, the non-parametric Kruskal-Wallis test (at significance level of P < 0.05) used. Finally, in case of existing differences between the groups and various demographic properties the Bonferroni post-hoc test have used.
