# Supplementary material for: The availability of health information system for decision-making with evidence-based medicine approach-a case study: Kermanshah, Iran
Source: Data Brief. 2018 May 24;19:890–5. doi: 10.1016/j.dib.2018.05.122 (PMC5997946; doi:10.1016/j.dib.2018.05.122)
Supplement: Supplementary file 1 — Supplementary material [file mmc1.docx]

Conflict of Interest

None of the authors of this paper have any conflicts of interest to declare.
